# Supplementary material for: Species Distribution, Antimicrobial Resistance, and Enterotoxigenicity of Non-aureus Staphylococci in Retail Chicken Meat
Source: Antibiotics (Basel). 2020 Nov 13;9(11):809. doi: 10.3390/antibiotics9110809 (PMC7697432; doi:10.3390/antibiotics9110809)
Supplement: Supplementary file 1 [file antibiotics-09-00809-s001.pdf]

antibiotics

MDPI

Table S1. Sequences of primers and PCR conditions used for the amplification of antimicrobial resistance, DNA gyrase, topoisomerase IV, and staphylococcal enterotoxin genes.

Target Genes	Primer Name	Sequences (5′→3′)	Amplicon Size (bp)	Ref.
mecA	mecA-F	GTAGAAATGACTGAACGTCCGATAA	310	[56]
	mecA-R	CCAATTCCACATTGTTTCGGTCTAA		
PCR conditions: 94 °C 5 min + 28 × (94 °C 30 s + 58 °C 45 s + 72 °C 1 min) + 72 °C 5 min				
ccr gene complex	ccrA1-609-628	CAAGCCTTATCAGGTACGAA		[57]
	ccrA2-1136-1155	CATTACGTCAACAACCGCAA		
	ccrA3-1106-1127	CTGAATCATTCAGAAAACAGAC		
	ccrA4-1121-1141	GTCCTAAAACAGCAACAAATGA		
	ccrB1-375-356	GAGCATTAACTTGCCTGTTG		
	ccrB2-72-51	CCTTCTGTGCTTTGCATTTC		
	ccrB3-223-204	GACCTTCGTTCGCATCTTTT		
	ccrB4-68-49	GGTTACAGTATTCAAGGTCAAT		
	ccrB6-505-481	CTGGTATGTTATTATATCCTAAAG		
	ccrC-181-202	GCAATGAAACGTCTATTACAAG		
	ccrC-382-361	CAAACATTGTAACGAGTACTTC		
PCR conditions: 94 °C 5 min + 28 × (94 °C 10 s + 56 °C 20 s + 72 °C 40 s) + 72 °C 5 min				
mec gene complex	mecA_univ-149-126	CTGCTATCTTTATAAACTTGTTTG		
	mecA_E-758-737	ACATAACCTAAAAGGTGTACTG		
	mecR1_B-987-966	TCATGTGAAGCTCGATATACT		
	mecR1_E-121-98	ACCAAACTTTATGATTTAACTGAG		
	mecR1_A-1110-1087	TTCATTATAAAGCACAAAACTTCC		
	IS431_C2-103-82	GTTGAATGATGAACGTTTACAC		
	IS431_C1-254-275	GGGACATACATTAGATATTTGG		
PCR condition: 94 °C 5 min + 28 × (94 °C 10 s + 56 °C 20 s + 72 °C 1 min) + 72 °C 5 min				
tet(K)	pAT102	TCGATAGGAACAGCAGTA	169	[58]
		CAGCAGATCCTACTCCTT		
tet(L)	pVB.A15	TCGTTAGCGTGCTGTCATTC	267	
		GATTCCCACCAATGTAGCCG		
tet(M)	pJ13	GTGGACAAAGGTACAACGAG	406	
		CGGTAAAGTTCGTCACACAC		
tet(O)	pUOA1	AACTTAGGCATTCTGGCTCAC	515	
		TCCCACTGTTCCATATCGTCA		
tet(S)	pAT451	CATAGACAAGCCGTTGACC	667	
		ATGTTTTTGGAACGCCAGAG		
PCR condition: 94 °C 5 min + 35 × (94 °C 1 min + 55 °C 1 min + 72 °C 1 min) + 72 °C 5 min				
gyrA	gyrA-F	AATGAACAAGGTATGACACC	368	this study
	gyrA-R	GCGATACCTGATGCACCATT		
PCR condition: 95 °C 5 min + 28 × (95 °C 30 s + 50 °C 30 s +72 °C 40 s) + 72 °C 5 min				
gyrB	gyrB-agnetis-F	TCTGAACTCTTTGAGCGTTT	456	
	gyrB-agnetis-R	AATATGTGCACCATCGACAT		
	PCR condition: 95 °C 5 min + 28 × (95 °C 30 s + 52 °C 30 s +72 °C 40 s) + 72 °C 5 min			
	gyrB-chromogenes-F	AGTTTACCAGGGAAGTTAGC	195	
	gyrB-chromogenes-R	AAATGCAGTGACCATTTGAC		
	PCR condition: 95 °C 5 min + 28 × (95 °C 30 s + 51 °C 30 s +72 °C 40 s) + 72 °C 5 min			
	gyrB-simulans-F	CCTCTCGTGCACGTATCGCA	300	
	gyrB-simulans-R	TGATATGCGCACCATCCACA		
	PCR condition: 95 °C 5 min + 28 × (95 °C 30 s + 48 °C 30 s +72 °C 40 s) + 72 °C 5 min			

	gyrB-lentus-F	AGAGCTCGTCTAGCAGCGAA	681	
	gyrB-lentus-R	CGTTTCGTCAGCTTCTATCGC		
	PCR condition: 95 °C 5 min + 28 × (95 °C 30 s + 54 °C 30 s +72 °C 40 s) + 72 °C 5 min			
parC	parC-agnetis-F	TTGGTGATCGATTTGGGCGT	180	
	parC -agnetis-R	CGTGGAGCCATTCACTAGCA		
	PCR condition: 95 °C 5 min + 28 × (95 °C 30 s + 54 °C 30 s +72 °C 40 s) + 72 °C 5 min			
	parC - chromogenes-F	ACTTGAAGATGTTTTAGGTGAT	459	
	parC - chromogenes-R	TTAGGAAATCTTGATGGCAA		
	PCR condition: 95 °C 5 min + 28 × (95 °C 30 s + 48 °C 30 s +72 °C 40 s) + 72 °C 5 min			
	parC -simulans-F	GAGTGCCAAAACAGTCGGTG	362	
	parC -simulans-R	TGTGCGGCGGAATATCTGTC		
	PCR condition: 95 °C 5 min + 28 × (95 °C 30 s + 54 °C 30 s +72 °C 40 s) + 72 °C 5 min			
	parC -lentus-F	ATCCAAGACCGAGCACTTCC	575	
	parC -lentus-R	CCGGTAGGGAAATCAGGTCC		
	PCR condition: 95 °C 5 min + 28 × (95 °C 30 s + 54 °C 30 s +72 °C 40 s) + 72 °C 5 min			
parE	parE-F	CGATTAAAGCACAACAAGCAAG	393	
	parE-R	GCGCACCATCAGTATCAG		
PCR condition: 95 °C 5 min + 28 × (95 °C 30 s + 48 °C 30 s +72 °C 40 s) + 72 °C 5 min				
sea	SEA-F	CAGCATACTATATTGTTTAAAGGC	400	
	SEA-R	CCTCTGAACCTTCCCATC		
sed	SED-F	CTAGTTTGGTAATATCTCCTTTAAACG	319	
	SED-R	TTAATGCTATATCTTATAGGGTAAACATC		
see	SEE-F	CAGTACCTATAGATAAAGTTAAAACAAGC	178	
	SEE-R	TAACTTACCGTGGACCCTTC		
PCR condition: 95 °C 3 min +33 × (95 °C 30 s + 53 °C 45 s + 72 °C 45 s) + 72 °C 5 min				
seb	SEB-F	GATTGGTGGTGTAACTGAGCA	351	
	SEB-R	TCAATCTTCACATCTTTAGAATCA		
sec	SEC-F	CTCAAGAACTAGACATAAAAGCTAGG	271	
	SEC-R	TCAAAATCGGATTAACATTATCC		
tst1	TSST-F	ATGGCAGCATCAGCTTGATA	350	
	TSST-R	TTTCCAATAACCACCCGTTT		
seg	SEG-F	AAGTAGACATTTTTGGCGTTCC	287	
	SEG-R	AGAACCATCAAACTCGTATAGC		
seh	SEH-F	GTCTATATGGAGGTACAACACT	213	
	SEH-R	GACCTTTACTTATTTCGCTGTC		
sei	SEI-F	GGTGATATTGGTGTAGGTAAC	454	[60,61]
	SEI-R	ATCCATATTCTTTGCCTTTACCAG		
selj	SEJ-F	ATAGCATCAGAACTGTTGTTCCG	152	
	SEJ-R	CTTTCTGAATTTTACCACCAAAGG		
sek	SEK-F	TAGGTGTCTCTAATAATGCCA	293	
	SEK-R	TAGATATTCGTTAGTAGCTG		
sell	SEL-F	TAACGGCGATGTAGGTCCAGG	383	
	SEL-R	CATCTATTTCTTGTGCGGTAAC		
sem	SEM-F	GGATAATTCGACAGTAACAG	379	
	SEM-R	TCCTGCATTAAATCCAGAAC		
sen	SEN-F	CATCATGCTTATACGGAGGAG	301	
	SEN-R	CCCACTGAACCTTTTACGTT		
seo	SEO-F	TGTGTAAGAAGTCAAGTGTAG	214	
	SEO-R	TCTTTAGAAATCGCTGATGA		
sep	SEP-F	TGATTTATTAGTAGACCTTGG	381	
	SEP-R	ATAACCAACCGAATCACCAG		
seq	SEQ-F	TCAAGGAGTTAGTTCTGGAAATT	251	
	SEQ-R	GCTTACCATTGACCCAGAGA		
ser	SER-F	GGATAAAGCGGTAATAGCAG	166	

	SER-R	GTATTCCAAACACATCTAAC	
selu	SEU-F	ATCAGAAACAAACATTAAAGCCCA	500
	SEU-R	TGACCATTTCCTTCGATAAACTTTAT	
PCR condition: 95 °C 3 min + 33 × (95 °C 30 s + 51 °C 45 s + 72 °C 45 s) + 72 °C 5 min			
